# Chk1 Promotes DNA Damage Response Bypass following Oxidative Stress in a Model of Hydrogen Peroxide-Associated Ulcerative Colitis through JNK Inactivation and Chromatin Binding

**DOI:** 10.1155/2017/9303158

**Published:** 2017-06-07

**Authors:** Kathrin Reissig, Andrew Silver, Roland Hartig, Antje Schinlauer, Diana Walluscheck, Thomas Guenther, Sandra Siedentopf, Jochen Ross, Diep-Khanh Vo, Albert Roessner, Angela Poehlmann-Nitsche

**Affiliations:** ^1^Department of Pathology, Otto-von-Guericke University, Magdeburg, Germany; ^2^Colorectal Cancer Genetics, Centre for Genomic and Child Health, Blizard Institute, Barts and The London School of Medicine and Dentistry, London, UK; ^3^Institute for Molecular and Clinical Immunology, Otto-von-Guericke University, Magdeburg, Germany; ^4^Department of Pathology, Hamburg, Germany; ^5^Academic Department of Histopathology, St. Mark's Hospital, Harrow, Middlesex, UK

## Abstract

Dysregulation of c-Jun *N*-terminal kinase (JNK) activation promoted DNA damage response bypass and tumorigenesis in our model of hydrogen peroxide-associated ulcerative colitis (UC) and in patients with quiescent UC (QUC), UC-related dysplasia, and UC-related carcinoma (UC-CRC), thereby adapting to oxidative stress. In the UC model, we have observed features of oncogenic transformation: increased proliferation, undetected DNA damage, and apoptosis resistance. Here, we show that Chk1 was downregulated but activated in the acute and quiescent chronic phases. In both phases, Chk1 was linked to DNA damage response bypass by suppressing JNK activation following oxidative stress, promoting cell cycle progression despite DNA damage. Simultaneously, activated Chk1 was bound to chromatin. This triggered histone acetylation and the binding of histone acetyltransferases and transcription factors to chromatin. Thus, chromatin-immobilized activated Chk1 executed a dual function by suppressing DNA damage response and simultaneously inducing chromatin modulation. This caused undetected DNA damage and increased cellular proliferation through failure to transmit the appropriate DNA damage signal. Findings in vitro were corroborated by chromatin accumulation of activated Chk1, Ac-H3, Ac-H4, and c-Jun in active UC (AUC) in vivo. Targeting chromatin-bound Chk1, GCN5, PCAF, and p300/CBP could be a novel therapeutic strategy to prevent UC-related tumor progression.

## 1. Introduction

Ulcerative colitis (UC) is an inflammatory bowel disease (IBD) characterized by recurrence-remission cycles with periods of mucosal ulceration accompanied by cell death and regeneration of the colonic mucosa. Little is known about the role of cell cycle arrest and its override in UC [1, 2]. The most important molecule involved in inflammation in UC is hydrogen peroxide (H_2_O_2_) [3]. Therefore, we established an experimental model of H_2_O_2_-associated colitis to investigate molecular mechanisms underlying UC [4, 5]. We generated the cellular in vitro model of UC by simulating its clinical course by exposing human colonic epithelial cells (HCEC) to repeated cycles (C) of H_2_O_2_ exposures. In this way, we generated ten C-cell cultures (C1-C10) following repeated H_2_O_2_ treatment interspersed with a recovery period which display the quiescent chronic phase of UC [4, 5]. A single H_2_O_2_ treatment of HCEC or C-cell cultures simulated the acute phase as well as the active chronic phase of UC, respectively [4, 5]. In this experimental model, we hypothesize that an ineffective JNK-dependent DNA damage checkpoint control in the acute phase was responsible for undetected DNA damage and increased proliferation in the quiescent chronic phase, both hallmarks of neoplastic transformation and cancer [6, 7]. Importantly, we showed an increase in the levels of intracellular reactive oxygen species (ROS) in C-cell cultures, suggesting that this intracellular oxidative stress serves as internal growth trigger [5]. Cellular survival was based on a switch from cell cycle arrest in the acute phase to increased proliferation in the quiescent chronic phase. This suggested that override of the upstream cell cycle arrest has an important function in UC and UC-associated neoplasia. Our recent in vivo data propose that JNK-dependent cell cycle arrest is critical in AUC, while chronic inflammation causes dysregulated JNK activity in QUC that contributed to checkpoint bypass, thus supporting our hypothesis [8]. In the experimental model, we further revealed a nonapoptotic function for the caspases: they can override the G1/S and intra-S checkpoints [4]. Moreover, dysregulated JNK activation, namely, p54 JNK inactivation and p46 JNK activation, and p21^WAF1^ downregulation caused further checkpoint adaptation through defective maintenance of the G2/M and mitotic spindle checkpoints [5].

The checkpoint kinase 1 (Chk1) is known for its role in DNA damage response, especially the G2/M and the mitotic spindle checkpoint, supporting the idea of an interplay between DNA damage and spindle checkpoints [9–15]. Activation of Chk1 through phosphorylation is realized through ataxia telangiectasia and Rad3 related (ATR) in response to DNA damage. Chk1 seems to protect against tumorigenesis, as deletion is associated with spindle checkpoint defects [16, 17] that cause chromosomal instability, aneuploidy, and eventually cancer [18], suggesting a tumor suppressive function for Chk1. Speroni and colleagues found that Chk1 promotes the progression of replication forks following DNA damage [19]. Recently, a novel epigenetic role of Chk1 was found that relates to its chromatin-binding ability and recruitment of the histone acetyltransferases (HATs) [20–22]. In undamaged cells, Chk1 is associated with chromatin and Chk1 dissociates from chromatin following its phosphorylation in response to DNA damage accompanied by loss of histone H3^T11^ phosphorylation [20, 23]. However, the function of Chk1 in UC tumorigenesis still needs to be fully elucidated.

Recently, we reported the override of the G1/S and intra-S checkpoints as well as the adaptation of the G2/M and mitotic spindle checkpoints in the experimental model of UC [4, 5]. In this study, we also link Chk1 to these checkpoint bypasses. We hypothesize that HCEC survive multiple H_2_O_2_ exposures through constitutively downregulated and activated Chk1, as well as through Chk1-dependent suppression of JNK-dependent DNA damage response. Simultaneously, activated Chk1 did not dissociate but remained associated with chromatin following oxidative stress. This triggered histone acetylation, binding of HATs, and transcription factors onto chromatin, and thus gene induction and proliferation. Here, we report that Chk1 executes a dual function in the experimental model of UC by suppressing DNA damage response and simultaneously inducing chromatin modulation; both these events trigger accumulated DNA damage and increased proliferation. We also observed accumulation of activated Chk1, Ac-H3, Ac-H4, and c-Jun on chromatin in AUC, which corroborates the importance of Chk1-mediated chromatin modulation in vivo.

## 2. Material and Methods

### 2.1. Cell Culture

HCEC were obtained from Professor Pablo Steinberg (Institute of Food Toxicology and Analytical Chemistry, University of Veterinary Medicine Hanover Germany).

C-cell cultures were generated as reported earlier [4, 5]. HCEC and C-cell cultures were cultured as described previously [4, 5].

### 2.2. Chk1 Knockdown

Chk1 knockdown was performed using the siRNA transfection technique according to the manufacturer's instructions (Santa Cruz Biotechnology, Santa Cruz, CA, USA) at a siRNA concentration of 20 nM and as described earlier [14].

### 2.3. Immunoblot Analysis

Proteins were prepared as described previously [24]. The following antibodies were used: JNK (#9258), p-JNK (Thr183/Tyr185) (#4668), c-Jun (#9165), p-c-Jun (Ser63) (#9261), Ac-H3^K9^ (#9649), Ac-lysine (#9814), p-H3^T11^ (#9764), PCAF (P300/CBP-associated factor) (#3378), GCN5 (#3305), and ATF2 (#9226) (Cell Signaling Technology, Danvers, MA, USA); p21^WAF1^ (OP64, Calbiochem, Darmstadt, Germany); *β*-actin (A5441, Sigma-Aldrich, Steinheim, Germany); Chk1 (sc-8408, Santa Cruz Biotechnology, Santa Cruz, CA, USA); Ac-H3 (#06-599), Ac-H4 (#06-866), H2AX (#07-627), and p300/CBP (CREB-binding protein) (#05-257) (Upstate, New York, NY, USA); p-Chk1 Ser317 (NB10092499, Novus Biologicals, Littleton, CO, USA); *γ*-H2AX (#05-636, Millipore, Darmstadt, Germany); and p300/CBP (NB120-3164, Acris, Herford, Germany). Densitometric analysis of the data was performed using the GeneTools Software from Syngene (Cambridge, UK). Fold induction (ratio protein/*β*-actin) was calculated by using the loading control *β*-actin. We regarded the regulation of proteins as follows: downregulated: ≤0.90, upregulated: ≥1.10, and unchanged: between >0.90 and <1.10.

### 2.4. 8-OHdG ELISA

Determination of 8-OHdG concentrations was performed using the Oxidative DNA Damage ELISA Kit from Cell Biolabs (San Diego, CA) according to their instructions.

### 2.5. Comet Assay

The alkaline comet assay was performed using the Kit from Trevigen (Gaithersburg, USA) as described previously [14].

### 2.6. Subcellular Fractionation

The subcellular fractionations of HCEC and C-cell cultures, as well as of human formalin fixed tissues, were performed using the Subcellular Protein Fractionation Kit from Thermo Scientific (Rockford, USA) according to the manufacturer's instructions and described previously [4]. Ponceau S served as the loading control. In the case of chromatin fractions, we used total histones stained by Ponceau S. In the case of analyzing proteins in nonchromatin fractions, we used the same region of Ponceau S-stained membrane as loading control.

### 2.7. Cell Cycle Analysis (FACS)

For flow cytometric analysis of DNA content and cell cycle phase distribution following H_2_O_2_ treatment or Chk1 knockdown, cells were collected 24, 48, and 72 h after treatment and fixed as described earlier [14]. Cell cycle analysis was performed as described previously using a flow cytometer (LSRFortessa, BD Bioscience, San Jose, CA) [4, 14]. Cell cycle distribution (percentage of cells) in cell debris (sub-G1) and G1, S, and G2/M phases of the cell cycle was analyzed using DIVA software v. 6.1.3 (BD Bioscience, San Jose, CA).

### 2.8. Statistical Analysis of Data

Results are expressed as mean ± SEM. Shapiro-Wilk and Student's *t*-tests were performed for data analysis with SigmaPlot 12 Software. Differences were considered as significant for *P* values of ≤0.5.

## 3. Results

### 3.1. Chk1 Is Downregulated but Activated in the Modelled Acute and Quiescent Chronic Phase of UC

**Figure 1:**
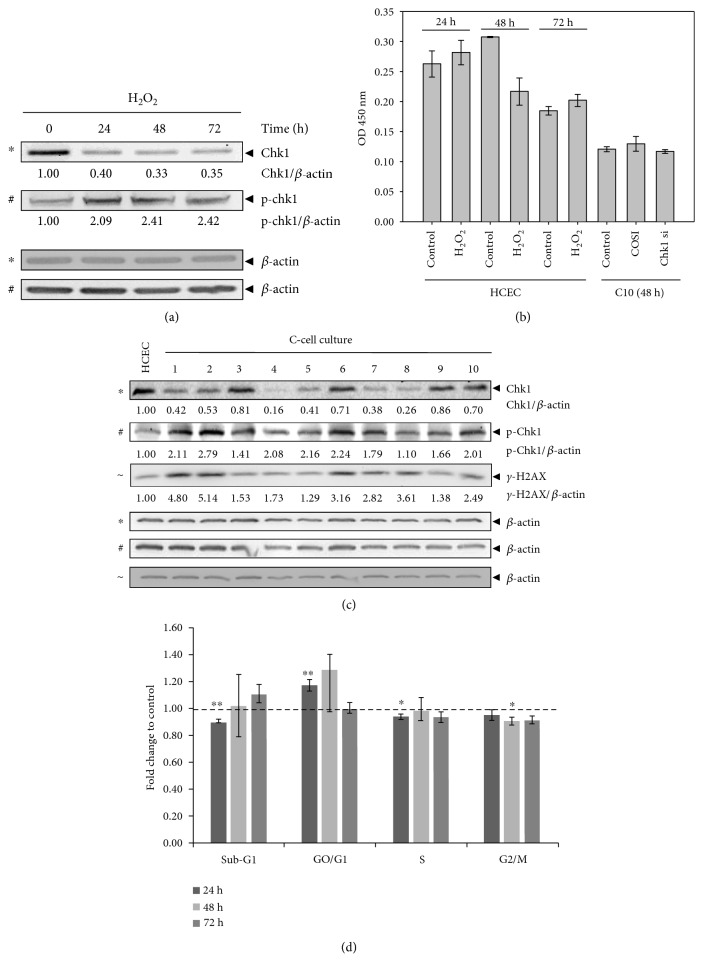
Chk1 is downregulated and activated in acute and quiescent chronic phase of experimental UC. (a) Immunoblot analysis of H_2_O_2_-treated HCEC. Lysates were analyzed by immunoblotting with Chk1, p-Chk1, and *β*-actin antibodies. *β*-actin served as loading control, and fold expression relative to HCEC is given below the blots. (b) Quantitative measurement of 8-OHdG in HCEC 24, 48, and 72 h after H_2_O_2_ treatment and in C10 cells 48 h following Chk1 siRNA transfection. (c) Immunoblot analysis of C-cell cultures. Lysates from C1-C10 cells and HCEC were immunoblotted with Chk1, p-Chk1, *γ*-H2AX, and *β*-actin antibodies. *β*-actin served as loading control, and fold expression relative to HCEC cells is given below the blots. *γ*-H2AX immunoblotting for C1 to C3 cells is already published in Poehlmann et al. [4]. (d) Cell cycle distribution of HCEC following Chk1 siRNA and control siRNA transfection and H_2_O_2_ treatment. The bar graphs represent the *x*-fold increase versus control siRNA as the mean ± S.E. of three independent experiments: ^∗^*P* < 0.5, ^∗∗^*P* < 0.01.

**Figure 2:**
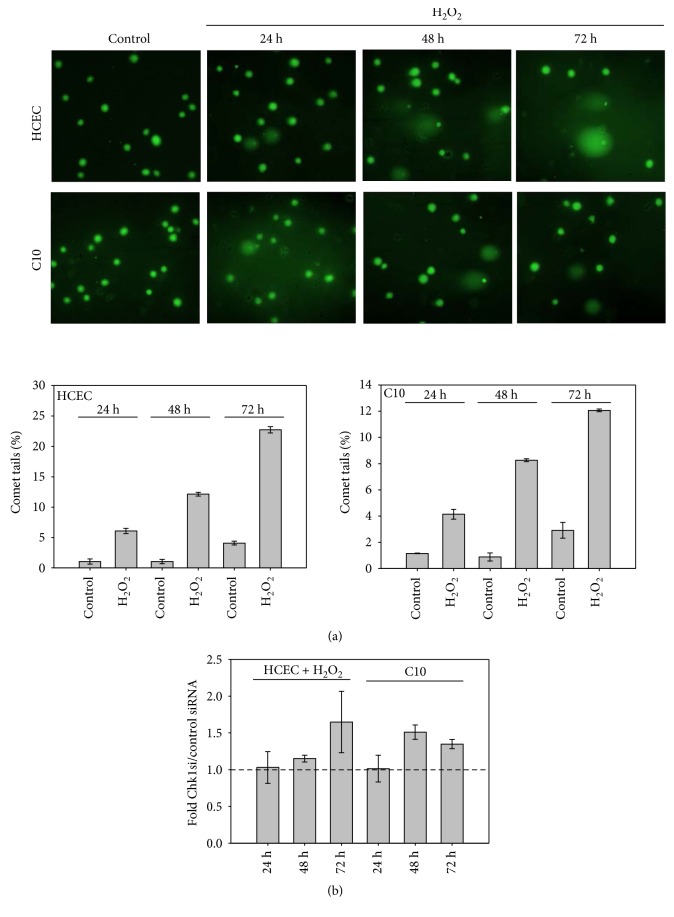
Comet assay analysis of H_2_O_2_-treated HCEC and C10 cells (a) and of C10 cells or H_2_O_2_-treated HCEC following Chk1 knockdown (b).

We recently reported that the dysregulation of JNK activity promotes DNA damage response bypass and tumorigenesis in both the experimental UC model [4, 5] and patients with QUC, UC-related dysplasia, and UC-CRC [8]. In the experimental model, JNK was activated in the acute phase, while upregulation of p-p46 JNK (C1-C10) and downregulation of p-p54 JNK (C3-C10) were observed in C-cell cultures [4, 5]. Based on the known role of Chk1 as a key regulator in DNA damage response and cell cycle progression, we analyzed its expression and activation in the acute and quiescent chronic phases of our UC cellular model. In the acute phase, treatment of HCEC with H_2_O_2_ led to Chk1 downregulation after 24 to 72 h, while activation of Chk1 through phosphorylation was observed over time (Figure 1(a)). Moreover, we detected the upregulation of the DNA damage sensor *γ*-H2AX that detects DNA single and double-stranded breaks (SSB, DSB) in H_2_O_2_-treated HCEC [4]. Furthermore, we observed comet tails in HCEC 24, 48, and 72 h after H_2_O_2_ treatment, further supporting the existence of SSB and DSB (Figure 2(a)). In addition, we measured oxidative DNA damage in HCEC by the formation of 8-hydroxydeoxyguanosine (8-OHdG), which is an ubiquitous marker of oxidative stress. We detected decreased absorbance at 450 nm 48 h after H_2_O_2_ treatment which reflects increased 8-OHdG formation (Figure 1(b)) while that 8-OHdG level accumulated at 72 h after H_2_O_2_. Untreated HCEC also clearly showed reduced absorbance at 450 nm after 72 h, which might be explained by intracellular ROS production in HCEC without H_2_O_2_ exposure over time [5]. Importantly, H_2_O_2_-exposed HCEC and C-cell cultures exhibited higher ROS generation relative to HCEC, supporting the hypothesis that exogenous H_2_O_2_ stimulated intracellular ROS production [5]. The observation that H_2_O_2_ did not further increase 8-OHdG formation after 72 h compared to HCEC control is indicative for sufficient base excision repair of damaged DNA by exonucleases in HCEC that were treated once with H_2_O_2_. *γ*-H2AX expression was still upregulated 72 h following H_2_O_2_ [4]_,_ which supports accumulation of DSB that was also shown by increased number of comet tails (Figure 2(a)). Importantly, we detected both constitutive downregulation and activation of Chk1 in quiescent chronic phase of UC (Figure 1(c)) which was paralleled by an increase of 8-OHdG of C10 cells as indicated by decreased absorbance (Figure 1(b)). We could not enhance this effect by further reducing the Chk1 level in C10 cells (Figure 1(b)).

Our recent in vivo data revealed a smaller increase in *γ*-H2AX levels in UC-CRC compared to colorectal cancer (CRC), which led us to propose that colitis-triggered inflammation masked DNA damage [8] as has been shown for C3 cells in vitro [4]. In this context, we did also not observe considerable upregulation of *γ*-H2AX in C4-C10 cells, except for C6 and C8 cells, compared to C1 and C2 cells (Figure 1(b)). This further supported the hypothesis that chronic inflammation reduces the sensitivity of the DNA damage signaling response, a prerequisite for DNA damage response bypass.

Further, we compared the extent of SSB and DSB in HCEC, C3, and C10 cells. We found significantly damaged DNA of C3 cells as shown by enlarged nuclei and comet tails, suggesting accumulated SSB and DSB in C3 cells [4]. Interestingly, C10 cells did not show significant comet tails or enlarged nuclei, indicating repair of SSB and DSB (Figure 2(a)). Moreover, we performed comet assay analysis of C10 cells and of H_2_O_2_-treated HCEC following Chk1 knockdown (Figure 2(b)). Reduced Chk1 level caused an increased number of comet tails in both cell lines, suggesting that Chk1 is required for sufficient DSB repair.

Overall, we found downregulation and activation of Chk1 in the acute phase of UC, while both are manifested constitutively in the quiescent chronic phase together with a lower than expected increase in *γ*-H2AX levels. This suggested that constitutive Chk1 downregulation and activation contribute to DNA damage response bypass and features of neoplastic transformation in the in vitro UC model.

### 3.2. Chk1 Promotes DNA Damage Response Bypass in the Acute Phase of Experimental UC

Our recent publication showed that H_2_O_2_ activates DNA damage checkpoints in HCEC by inducing JNK-dependent S- and G2/M cell cycle arrests, while nonapoptotic caspases simultaneously mediated an override of the G1/S and intra-S checkpoints, thereby bypassing DNA damage response and promoting G1 and S phase progression despite DNA damage [4]. Here, we investigated whether Chk1 affected cell cycle progression in the acute phase. As shown for inhibited caspase activity [4], decreased S cell population and a significantly increased G1 cell population was also detected 24 h after H_2_O_2_ treatment of Chk1-siRNA transfected HCEC (*P* = 0.004, Figure 1(d)). In contrast, the sub-G1 cell population was significantly decreased after 24 h (*P* = 0.002) but then increased after 72 h. Therefore, HCEC accumulated in G1 following Chk1 knockdown, while they subsequently underwent apoptosis. Thus, Chk1 seems to promote progression of cells through G1 and S phase via bypassing the G1/S and intra-S checkpoints. At later time points (48 and 72 h), we observed fewer cells in the G2/M phase (Figure 1(d)). Therefore, Chk1 mediated cellular survival by halting cells in the G2/M phase. Thus, damaged cells may enter mitosis following Chk1 downregulation. In this context, a higher percentage of cells underwent apoptosis. Thus, damaged cells slipped through mitosis, re-entered the cell cycle in G1, and underwent apoptosis induced by the G1-tetraploidy checkpoint or damaged cells underwent mitotic catastrophe as reported earlier [14]. This supports the proposal that Chk1 mediated survival and mitotic checkpoint control.

In summary, Chk1 promoted cell cycle progression and hence survival following oxidative stress by DNA damage response bypass by assisting progression of cells through G1 and S phase and the halting of cells in the G2/M phase.

### 3.3. Chk1 Is Linked to DNA Damage Response Bypass by Suppressing JNK Activation and *γ*-H2AX Expression in the Modelled Acute Phase of UC

**Figure 3:**
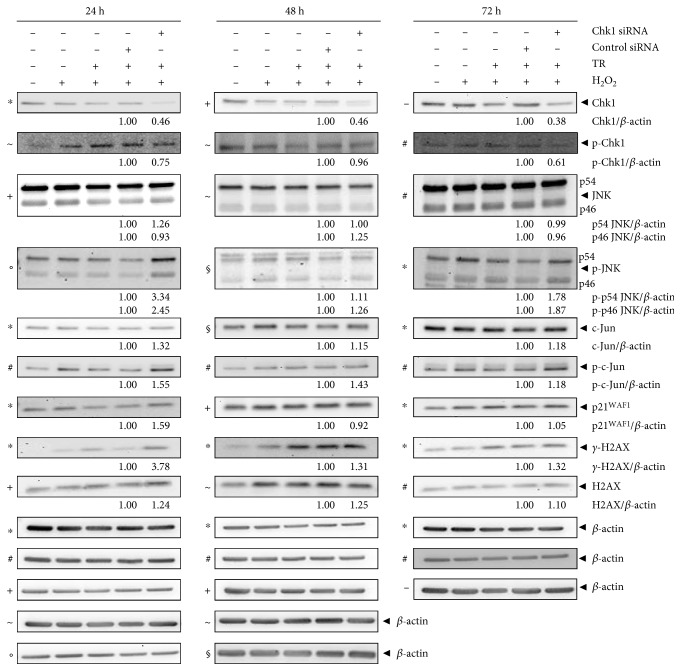
Chk1 promotes DNA damage response bypass by suppressing JNK activation. Immunoblot analysis of Chk1 siRNA-transfected and H_2_O_2_-treated HCEC 24, 48, and 72 h after H_2_O_2_ treatment. Lysates were analyzed by immunoblotting with Chk1, p-Chk1, JNK, p-JNK, c-Jun, p-c-Jun, p21^WAF1^, *γ*-H2AX, H2AX, and *β*-actin antibodies. *β*-actin served as loading control as marked (−, ∗, +, #, ◦, ~, and §), and fold expression relative to control siRNA is given below the blots.

HCEC respond to DNA damage through the activation of JNK and c-Jun, which, in turn, led to p21^WAF1^, *γ*-H2AX, and caspase upregulation [4]. Importantly, caspases were found to suppress *γ*-H2AX and p21^WAF1^ upregulation as the first response to DNA damage and JNK and c-Jun activation as the second response [4]. To further assess the role of Chk1 in this DNA damage response bypass, we performed immunoblot analyses of proteins of the JNK pathway following Chk1 knockdown (Figure 3). We found upregulation of p-p54 JNK, p-p46 JNK, c-Jun, p-c-Jun, *γ*-H2AX, and H2AX after 24 to 72 h. p54 JNK and p46 JNK expression was increased after 24 h or 48 h, respectively. Interestingly, p21^WAF1^ was increased after 24 h but was nearly unchanged after 48 and 72 h. Thus, Chk1 did not suppress p21^WAF1^ further during recovery from oxidative stress. Although Chk1 levels decreased following Chk1 knockdown, the level of activated Chk1 remained unchanged 48 h after knockdown.

On the basis of these results, we concluded that JNK activation was suppressed by Chk1, including p-p46 JNK and p-p54 JNK, which also resulted in the repressed expression of the JNK signaling molecules c-Jun, p-c-Jun, p21^WAF1^, *γ*-H2AX, and H2AX. This makes the JNK pathway ineffective for DNA damage checkpoint control in the acute phase of the UC experimental model and enhances DNA damage response bypass caused by Chk1 downregulation itself. We propose that Chk1 prevents proper G1/S and intra-S checkpoint activation through negative regulation of JNK activation and of expression of the JNK-regulated proteins p21^WAF1^ and *γ*-H2AX, as has also been shown for the nonapoptotic caspases [4]. In addition, H_2_O_2_ signaling following Chk1 knockdown was transient with activation of proteins such as p-JNK, p-c-jun, p21^WAF1^, and *γ*-H2AX with their induction as the first response and their decline as the second response (72 h), which is a characteristic sign of recovery from oxidative DNA damage [4].

### 3.4. Chk1 Also Suppresses JNK Activation and *γ*-H2AX Expression in the Quiescent Chronic Phase of Experimental UC

**Figure 4:**
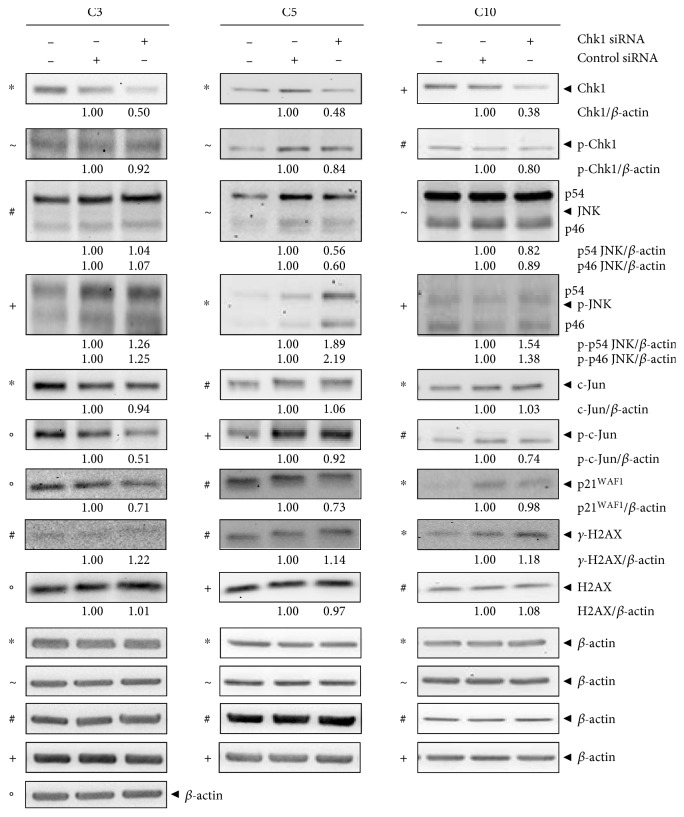
Chk1 is a negative regulator of JNK activation in quiescent chronic phase of experimental UC. Immunoblot analysis of Chk1 siRNA-transfected C3, C5, and C10 cells 24 h after transfection. Lysates were analyzed by immunoblotting with Chk1, p-Chk1, JNK, p-JNK, c-Jun, p-c-Jun, p21^WAF1^, *γ*-H2AX, H2AX, and *β*-actin antibodies. *β*-actin served as loading control as marked (∗, +, #, ◦, and ~), and fold expression relative to control siRNA is given below the blots.

Next, we analyzed whether Chk1 also contributed to the override of cell cycle checkpoint control in the modelled quiescent chronic phase of UC. We performed immunoblot analysis of C3, C5, and C10 cells 24 h following Chk1 knockdown (Figure 4). Indeed, we detected upregulated p-p46 JNK, p-p54 JNK, and *γ*-H2AX in C3, C5, and C10 cells following Chk1 knockdown. In C3 cells, p-c-Jun and p21^WAF1^ were downregulated and the levels of activated Chk1, p46 JNK, p54 JNK, c-Jun, and H2AX were unaffected by Chk1 knockdown. We observed downregulation of p46 JNK, p54 JNK, and p21^WAF1^ in C5 cells, while c-Jun, p-c-Jun, and H2AX levels were unchanged following Chk1 knockdown. In C10 cells, we found downregulated p46 JNK, p54 JNK, and p-c-Jun. Expression of c-Jun, p21^WAF1^, and H2AX was unchanged following Chk1 knockdown.

In summary, we observed (i) Chk1-dependent suppression of p-p46 JNK, p-p54 JNK, and *γ*-H2AX in C3, C5, and C10 cells and (ii) Chk1-dependent induction of p-c-Jun and p21^WAF1^ in C3 cells, of p21^WAF1^ in C5 cells, and of p-c-Jun in C10 cells. Thus, Chk1 contributed to inefficient checkpoint control and the proliferative response in the modelled quiescent chronic phase of UC, through keeping the JNK pathway ineffective by Chk1-dependent p-JNK and *γ*-H2AX suppression.

### 3.5. Chk1 Suppresses p46 JNK Activation and DNA Damage Response in the Modelled Active Chronic Phase of UC

**Figure 5:**
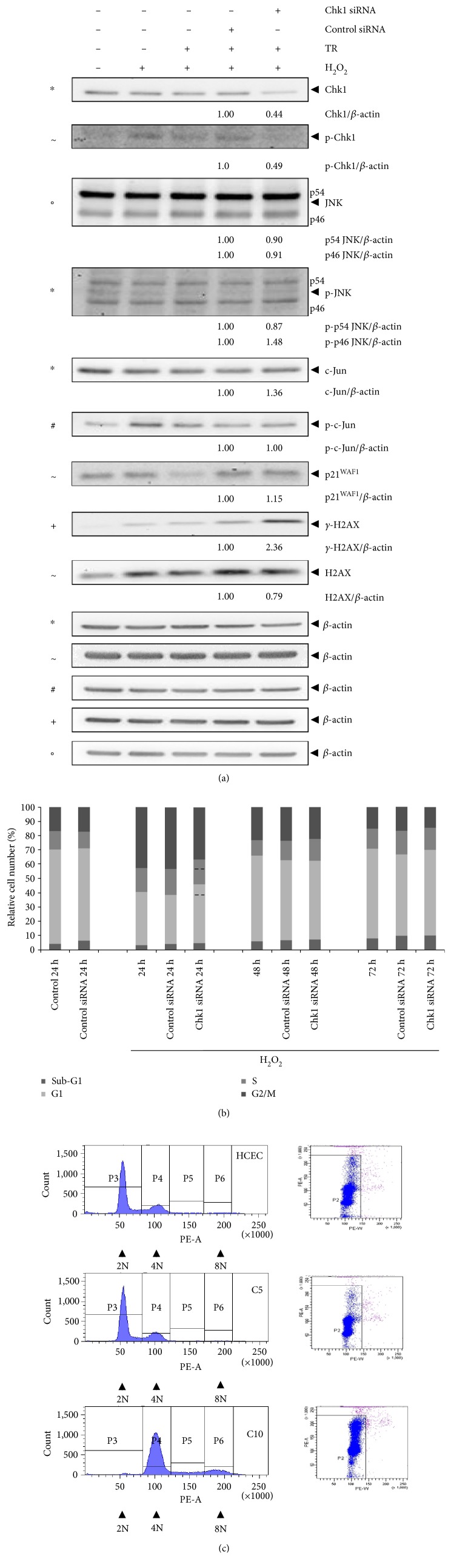
Chk1 downregulation contributes to defective maintenance of G2/M and mitotic spindle checkpoints. (a) Immunoblot analysis of Chk1 siRNA-transfected and H_2_O_2_-treated C10 cells 24 h after H_2_O_2_ treatment. Lysates were analyzed by immunoblotting with Chk1, p-Chk1, JNK, p-JNK, c-Jun, p-c-Jun, p21^WAF1^, *γ*-H2AX, H2AX, and *β*-actin antibodies. *β*-actin served as loading control as marked (∗, +, #, ◦, and ~), and fold expression relative to control siRNA is given below the blots. (b) Cell cycle distribution of C10 cells following Chk1 siRNA and control siRNA transfection and H_2_O_2_ treatment. Dashed lines contribute to cell cycle analysis with control siRNA transfection to mark increased G1 cell population and reduced G2/M arrest following Chk1 siRNA transfection. (c) Cell cycle distribution of HCEC, C5, and tetraploid C10 cells that were cultured under acidic conditions (10% CO_2_).

We observed upregulated p-p46 JNK, c-Jun, p21^WAF1^, and *γ*-H2AX; downregulated p54 JNK, p-p54 JNK, and H2AX; unaffected p46 JNK; and p-c-Jun in Chk1 siRNA-transfected and H_2_O_2_-treated C10 cells after 24 h (Figure 5(a)). In our recent publication, we reported that G2/M-arrested C10 cells entered mitosis and re-entered the cell cycle in the G1 phase instead of going into apoptosis because of an adapted G2/M and mitotic spindle checkpoint [5]. Cell cycle analysis following H_2_O_2_ treatment of Chk1 siRNA-transfected C10 cells showed more cells in the G1 phase after 24 and 72 hrs and fewer cells in G2/M phase after 24 h (Figure 5(b)). Thus, G2/M arrest in C10 cells seems to be partially mediated by Chk1 as in HCEC (Figure 1(d)). From this G2/M arrest, C10 cells did not enter apoptosis as shown for HCEC [4] but slip through mitosis and accumulate in G1 (Figure 5(b)). Thus, further Chk1 downregulation in C10 cells led to an enhanced override of the G2/M and mitotic spindle checkpoint, and apoptosis-resistant cells accumulate in the G1 phase.

In summary, we suggest that the recently reported defective maintenance of G2/M and mitotic spindle checkpoints that lead to apoptosis resistance [5] is caused by Chk1 downregulation in C-cell cultures.

### 3.6. Acidosis Induces Tetraploidy in Checkpoint-Defective C10 Cells

As described in our recent publication [5], and shown here (Figure 5(b)), Chk1 downregulated C10 cells override the G1/S, intra-S, G2/M, and the mitotic spindle checkpoints. This could potentially lead to tetraploidy, a consequence of chromosomal instability, particularly if the G1 tetraploidy checkpoint is also ineffective [25]. We therefore aimed to select tetraploid C10 cells by overriding the G1 tetraploidy checkpoint through inducing acidic conditions by increasing the CO_2_ concentration to 10% [26]. HCEC and C5 cells served as controls and were also incubated with 10% CO_2_. Next, we analyzed the DNA content of HCEC, C5, and C10 cells using flow cytometry, which revealed an almost complete loss of the diploid DNA content in C10 cells (Figure 5(c)). In parallel, the tetraploid DNA content was strongly increased. Furthermore, we observed an octaploid DNA content in C10 cells only; HCEC and C5 cells showed the expected normal distribution of diploid and tetraploid DNA. We further analyzed the tetraploid DNA content of C10 cells by increasing passage number. In C10 cells, tetraploidy has manifested in passages 17 to 42, with 4N values between 45% and 70% (Figure S1 available online at https://doi.org/10.1155/2017/9303158). In contrast, tetraploidy was not induced in HCEC and C5 cells, despite prolonged cultivation under acidic conditions.

These data support a dysfunction of the mitotic spindle checkpoint in checkpoint-defective C10 cells [27], leading to the generation of tetraploid cells, which is considered to be an important event in UC tumorigenesis [28].

### 3.7. Increased Histone Acetylation and Expression of Epigenetically Relevant Proteins in the Quiescent Chronic Phase of the Experimental UC Model

**Figure 6:**
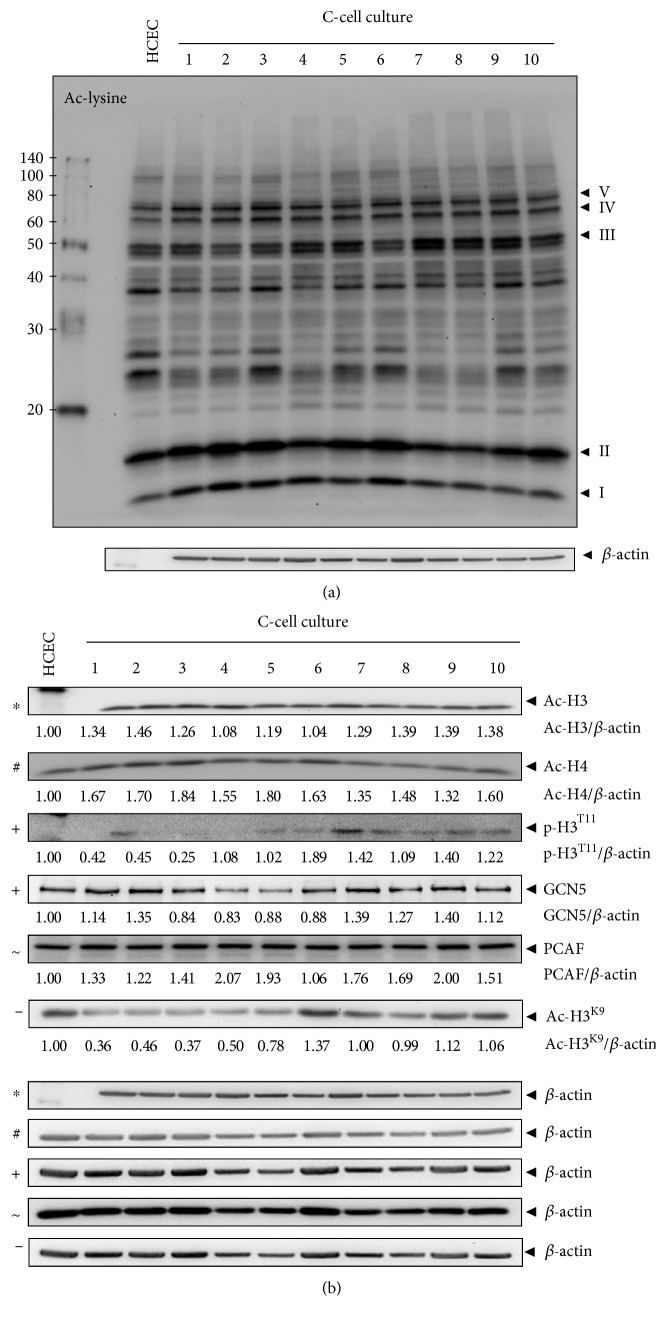
Increased expression of acetylated proteins and histone acetyltransferases in quiescent chronic phase. Immunoblot analysis of C1-C10 cells. Lysates were analyzed by immunoblotting with Ac-lysine and *β*-actin antibodies (a) or with Ac-H3, Ac-H4, p-H3^T11^, GCN5, PCAF, Ac-H3^K9^, and *β*-actin antibodies (b). *β*-actin served as loading control as marked (∗, +, #, ~, and −), and fold expression relative to HCEC is given below the blots.

Besides checkpoint control, Chk1 also functions in chromatin modulation through modification of histone acetylation [20]. We already showed the upregulation of the transcription factor ATF2 with an intrinsic HAT activity [29, 30] in C2 and C3 cells [5]. Here, we further investigated the epigenetic regulation of C-cell cultures via acetylation by analyzing the expression of acetylated histones and HATs such as GCN5 and PCAF. Lysine acetylation of histones, transcription factors, and other proteins affect chromatin structure, gene activity, and cell growth [31]. Proteins that are acetylated at the epsilon-amino group of lysine residues, especially in the range between 10 and 80 kDa (I–V), were found to be upregulated in C-cell cultures (Figure 6(a)). Both Ac-H3 and Ac-H4 were upregulated in C1-C10 cells, except for C4 and C6 cells for Ac-H3 (Figure 6(b)). Importantly, phosphorylation of histone H3 at Thr11 is Chk1-dependent [20] and was increased in C6, C7, C9, and C10 cells (Figure 5(b)). Moreover, PCAF was upregulated in all C-cell cultures, excluding C6, while increased GCN5 levels were found in C1, C2, and C7-C10 cells (Figure 6(b)). H3 acetylated at K9 was upregulated in C6, C9, and C10 cells. The detected increase in the overall expression of histone acetylation and HATs suggests epigenetic regulation of gene expression upon chronic DNA damage in our UC model as has also been shown for the oxidative stress-based therapy in colorectal cancer cells [24].

### 3.8. Increased Binding of Activated Chk1 on Chromatin in the Experimental Model of the Acute Phase of UC

**Figure 7:**
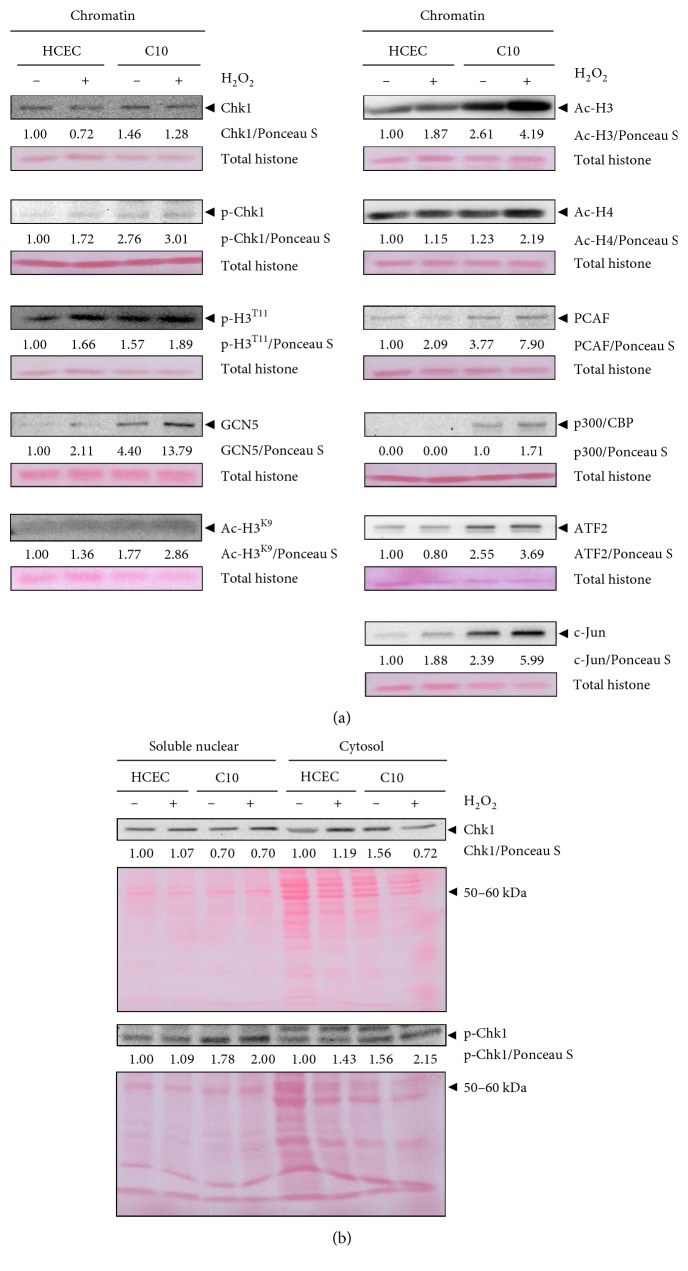
Acute and chronic DNA damage modulates Chk1 chromatin binding in vitro. (a) HCEC, H_2_O_2_-treated HCEC, C10 cells, and H_2_O_2_-treated C10 cells were fractionated, and chromatin-bound extracts were analyzed by immunoblotting using Chk1, p-Chk1, p-H3^T11^, GCN5, Ac-H3^K9^, Ac-H3, Ac-H4, PCAF, p300/CBP, ATF2, and c-Jun antibodies. Ponceau S staining served as loading control, and fold chromatin binding relative to HCEC is given below the blots. (b) Soluble nuclear and cytoplasmic extracts were analyzed by immunoblotting using Chk1 and p-Chk1 antibodies. Ponceau S staining served as loading control, and fold chromatin binding relative to HCEC is given below the blots.

We next investigated the role of Chk1 in chromatin modulation and analyzed the chromatin fractions of H_2_O_2_-treated and untreated HCEC following subcellular fractionation (Figure 7(a)). Although, we detected dissociation of Chk1 from chromatin of H_2_O_2_-treated HCEC, we could not detect chromatin dissociation of its phosphorylated form. Instead, activated Chk1 together with p-H3^T11^, GCN5, Ac-H3^K9^, Ac-H3, Ac-H4, PCAF, and c-Jun accumulated on chromatin of H_2_O_2_-treated HCEC. Interestingly, ATF2 dissociated from chromatin.

These data led us to propose that unexpected failed dissociation of activated Chk1 and the accumulation of HATs and the transcription factor c-Jun on chromatin of H_2_O_2_-treated HCEC caused restrained DNA damage response in the acute phase.

### 3.9. Activated Chk1, HATs, Acetylated Histones, and Transcription Factors Are Associated with Chromatin in the Quiescent Chronic Phase of Experimental UC

Next, we studied chromatin modulation on isolated chromatin from C10 cells following subcellular fractionation (Figure 7(a)). We detected enhanced chromatin association of Chk1 followed by increased binding of activated Chk1 on chromatin of C10 cells compared to HCEC and H_2_O_2_-treated HCEC. This was paralleled by unchanged level of pH3^T11^ compared to H_2_O_2_-treated HCEC. We also detected increased binding of GCN5, as well as that of Ac-H3^K9^ and Ac-H3 compared to HCEC and H_2_O_2_-treated HCEC. Other HATs as well as transcription factors also showed elevated binding to the chromatin of C10 cells, such as PCAF and ATF2, and c-Jun, respectively (Figure 7(a)). Interestingly, p300/CBP is only recruited to the chromatin following chronic oxidative stress. Furthermore, we also detected an enhanced level of activated Chk1 in the cytosolic fraction of C10 cells (Figure 7(b)), which could be associated with increased proliferation [32].

In summary, chronically induced accumulation of Chk1, reinforced binding of activated Chk1, GCN5, Ac-H3^K9^, PCAF, ATF2, and c-Jun, restrained accumulation of p-H3^T11^, and induced binding of p300/CBP on chromatin could induce the expression of genes known to cause uncontrolled proliferation and undetected DNA damage through DNA damage response bypass, which are hallmarks of cancer.

### 3.10. Reinforced Histone Acetylation and Binding of HATs and Transcription Factors on Chromatin in the Modelled Active Chronic Phase of UC

H_2_O_2_ treatment of C10 cells led to enhanced chromatin binding of GCN5, Ac-H3^K9^, Ac-H3, Ac-H4, PCAF, p300/CBP, ATF2, and c-Jun (Figure 7(a)) compared to HCEC, H_2_O_2_-treated HCEC, and C10 cells. Instead, chromatin binding of Chk1, activated Chk1, and p-H3^T11^ remained nearly unchanged. In parallel, we observed cell cycle arrest and its override, apoptosis resistance, and DNA damage response bypass which could be a consequence of the maintained chromatin-binding capacity of Chk1 and activated Chk1, and the increased capacity of HATs, acetylated histones, and transcription factors to bind on chromatin.

### 3.11. Chromatin-Bound Activated Chk1 Recruits Histone Acetyltransferases to Chromatin

**Figure 8:**
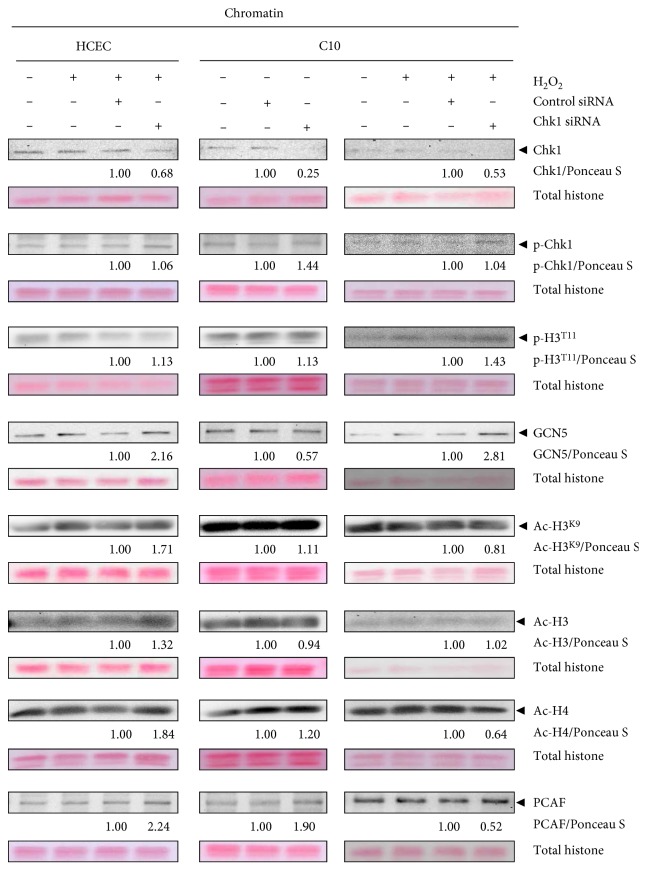
Acute, quiescent chronic, and active chronic oxidative stress induce chromatin modulation. HCEC and C10 cells were transfected with Chk1 siRNA or control siRNA, treated with H_2_O_2_, and subcellular fractionated. Chromatin-bound extracts were analyzed by immunoblotting using Chk1, p-Chk1, p-H3^T11^, GCN5, Ac-H3^K9^, Ac-H3, Ac-H4, and PCAF antibodies. Ponceau S staining served as loading control, and fold chromatin binding relative to control siRNA is given below the blots.

Moreover, we also performed chromatin-binding studies in untreated and H_2_O_2_-treated HCEC and C10 cells following Chk1 knockdown (Figure 8). Although chromatin-bound Chk1 level decreased following Chk1 knockdown, activated Chk1 remained unchanged chromatin-bound in H_2_O_2_-treated HCEC and C10 cells and showed even increased chromatin binding in untreated C10 cells. This further suggests that cells compensate reduced Chk1 level with maintaining high chromatin binding of activated Chk1, thus bypassing DNA damage response. Moreover, in Chk1 siRNA-transfected and H_2_O_2_-treated HCEC, we detected increased chromatin-bound p-H3^T11^, GCN5, Ac-H3^K9^, Ac-H3, Ac-H4, and PCAF. In C10 cells, the level of chromatin-bound GCN5 was reduced, those of p-H3^T11^, Ac-H3^K9^, Ac-H4, and PCAF were increased, but the level of Ac-H3 remained unchanged following Chk1 knockdown. In H_2_O_2_-treated C10 cells, we detected decreased levels of chromatin-bound Ac-H3^K9^, Ac-H4, and PCAF; unchanged binding of Ac-H3; but increased levels of p-H3^T11^ and GCN5 following Chk1 knockdown.

On the basis of these results, we propose that reduced chromatin-bound Chk1 resulted in the maintained binding of activated Chk1. This triggered the recruitment (i) of GCN5 and PCAF to chromatin in the acute phase, (ii) of PCAF to chromatin in the quiescent chronic phase, and (iii) of GCN5 to chromatin in the active chronic phase.

### 3.12. Activated Chk1, Ac-H3, Ac-H4, and c-Jun Are Bound to Chromatin in AUC In Vivo

**Figure 9:**
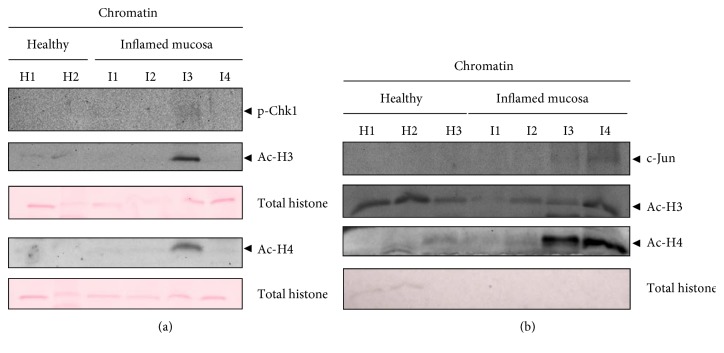
Activated Chk1, Ac-H3, Ac-H4, and c-Jun are chromatin-bound in AUC in vivo. Normal healthy colorectal mucosa (H1, H2, and H3) and inflamed tissue (I1–I4) was fractionated, and the chromatin-bound fractions were analyzed with p-Chk1, Ac-H3, and Ac-H4 antibodies (a) and with c-Jun, Ac-H3, and Ac-H4 antibodies (b). Ponceau S staining served as loading control. Localisations (a): healthy colorectal mucosa: H1, ascending colon; H2, descending colon; inflamed mucosa: I1, ascending colon; I2, ascending colon; I3, transverse colon; I4, descending colon. Localisations (b): healthy colorectal tissue: H1, ileum; H2; cecum; H3, ascending colon; inflamed mucosa: I1, transverse colon; I2, descending colon; I3, sigmoid colon; I4, rectum.

Next, we fractionated human tissues of inflamed mucosa of AUC patients. Importantly, we have found activated Chk1 together with Ac-H3 and Ac-H4 in one chromatin fraction of inflamed mucosa (I3: transverse colon) but not in noninflamed mucosa (Figure 9(a)). Hence, acetylated histones accumulated only in the p-Chk1-positive fraction. In addition, we also found chromatin-bound Ac-H3 and Ac-H4 together with c-Jun in two chromatin fractions of inflamed mucosa (I3: sigmoid colon, I4*:* rectum) (Figure 9(b)). Ac-H3 could also be detected in healthy colorectal mucosa of the same patient (H1-H3). Importantly, Ac-H4 accumulated only in the c-Jun-positive fractions. Overall, we suggest that chromatin binding of activated Chk1 and the oncoprotein c-Jun and subsequent histone acetylation is also an important mechanism in vivo.

## 4. Discussion

Chk1 is known for its various functions in DNA damage response, which are mostly generated by replication stress. This suggests that Chk1 could act as a potential tumor suppressor by restraining cell cycle progression and prevention of replication stress-associated genomic instability. Thus, Chk1 downregulation would contribute to genomic instability that ultimately leads to cellular transformation [33]. Recently, Lunardi et al. showed transcriptional repression of Chk1 through oncogenic E26 transformation-specific (ETS) transcription factors in prostate cancer, which resulted in reduced Chk1 levels and unrepaired DNA damage [34]. Moreover, DNA damage induces dissociation of activated Chk1 from chromatin, thereby reducing phosphorylation of H3^T11^, resulting in the decreased levels of chromatin-bound GCN5 and Ac-H3^K9^, which led to transcriptional repression [21, 23].

Because all of the DNA damage checkpoints in a recently developed experimental model of UC were shown to be JNK-dependent [4, 5], there is a strong possibility that this is also important in vivo [8]. Hence, the investigation of Chk1's role in DNA damage response bypass in the acute and chronic phase of the UC model is presented here. Interestingly, we noted constitutive Chk1 downregulation and activation, Chk1-dependent suppression of JNK activation, and an unexpected binding of activated Chk1 on chromatin in the acute and the quiescent and active chronic phase of the UC model. This should abrogate transcriptional repression, promoting DNA damage response bypass and features of oncogenic transformation.

### 4.1. Chk1 Drives Progression of Cells through the Cell Cycle in the Modelled Acute Phase of UC through Suppression of the JNK Pathway

Accumulating evidence suggests that Chk1 may promote rather than suppress tumor growth [35]. We found Chk1 downregulation but prolonged activation of Chk1 in the recovery from DNA damage in HCEC. Indeed, as reported for the nonapoptotic function of caspases, we observed that also Chk1 was involved in mediating survival of oxidatively damaged HCEC through suppression of the JNK pathway. In detail, we observed Chk1-dependent suppression of JNK activation (p46 and p54 JNK) and JNK signaling (repressed c-Jun, p-c-Jun, p21^WAF1^, *γ*-H2AX, and H2AX), which promoted G1/S and intra-S cell cycle progression, contributing to features of neoplastic transformation. This survival mechanism in the acute phase is linked to increased proliferation and undetected DNA damage in quiescent chronic phase. In the context of cancer therapy, downregulated Chk1 causes checkpoint abrogation, thereby potentiating the toxicity of 5-fluorouracil by apoptosis induction in human cervical and lung cancer cells [36].

Overall, Chk1 was shown to suppress JNK activation and signaling, including the repression of c-Jun, p-c-Jun, *γ*-H2AX, H2AX, and p21^WAF1^ in the acute phase, promoting progression of cells through G1 and S phase following circumvention of DNA damage checkpoint control.

### 4.2. Chk1 Downregulation and Activation Have Manifested in the Modelled Quiescent Chronic Phase of UC

Moreover, a sustained activated Chk1 level was also found in quiescent chronic phase, while Chk1 was further downregulated. We hypothesize that both constitutive changes might drive cell cycle progression and accumulated DNA damage, which follow a previous JNK-dependent cell cycle arrest. We conclude that the signaling pathways responsible for these changes are manifested in the quiescent chronic phase. However, the fluctuation in signaling protein expression of C-cell cultures may be explained by the fact that we detected both the DNA damage response and the adaptation to the DNA damage response, which may counteract the ROS-induced changes, and this may vary in between C-cell cultures.

Additionally, we propose that masked DNA damage in C-cell cultures causes the cell signal machinery to provide signals that cells use to re-enter the cell cycle. In this context, activated Chk1 supports re-entry into the cell cycle following stalled replication [37]. On the other hand, Wang et al. reported that constitutively activated Chk1 may represent a novel strategy to suppress tumor growth [38]. However, the cellular localisation of Chk1 is critical for checkpoint response and cell viability [39]. We propose that cells compensate for low Chk1 level by reinforcing Chk1 activation, while activated Chk1 remains chromatin-bound.

### 4.3. Chk1 Pushes Cells over the Checkpoints in Modelled Quiescent and Active Chronic Phase of UC through Suppression of JNK Activation

We observed Chk1-dependent suppression of JNK activation and *γ*-H2AX expression in C3, C5, and C10 cells. Moreover, induction of p21^WAF1^ via Chk1 was observed in C3 and C5 cells, which seems to be JNK-independent due to its Chk1-mediated suppression. Further downregulation of the decreased Chk1 level in C-cell cultures showed that *γ*-H2AX induction and therefore detection of DNA damage is linked to JNK activation, while p21^WAF1^ is not induced upon JNK activation following Chk1 knockdown. Thus, suppression of p21^WAF1^ in C-cell cultures is not mediated through Chk1, while nonapoptotic caspases were found to be responsible [4]. Importantly, Chk1 seems to be a further player in altering JNK activation in C-cell cultures to decrease DNA damage response that can drive cells to neoplastic transformation. Thereby, Chk1 suppresses activation of p46 JNK and p54 JNK such as the nonapoptotic caspases [4]. Thus, another mechanism seems to be responsible for the observed p46 JNK activation in C-cell cultures [5].

Based on our data, the question arises on how Chk1 suppresses JNK activation based on the assumption that suppression is mediated through Chk1 kinase activity. The observed high levels of activated Chk1 following Chk1 knockdown indicates that Chk1-dependent suppression of JNK activation is independent of Chk1 kinase activity. However, we cannot exclude that activated Chk1 suppressed JNK activation. These data further support the idea that chronically irritated cells compensate low Chk1 level by reinforcing Chk1 activation, while activated Chk1 is chromatin-bound.

Interestingly, we observed Chk1-dependent suppression of p46 JNK activation and of expression of c-Jun, p21^WAF1^, and *γ*-H2AX in H_2_O_2_-treated C10 cells. Moreover, H_2_O_2_ treatment of Chk1 siRNA-transfected C10 cells caused enhanced override of the G2/M and mitotic spindle checkpoints, resulting in apoptosis-resistant cells that accumulate in the G1 phase. Importantly, further stressing checkpoint-defective C10 cells using acidic conditions induced tetraploidy, which is an important tumorigenic event in UC carcinogenesis [27].

### 4.4. Chk1 Downregulation Is Linked to DNA Damage Response Bypass

The DNA damage response consists of the base excision and DSB repair, while overcoming this response may lead to genetic instability. Moreover, a failure in the mitotic checkpoint may cause also chromosomal instability. *γ*-H2AX is associated with both chromosomal stability and DSB repair [40]. As we did not observe accumulation of SSB and DSB in C10 cells through the comet assay, we concluded effective DSB repair. Thus, repetitive DNA damage-induced chromatin changes may promote DNA repair and genomic stability, which was also reported by Luijsterburg and van Attikum [41]. We detected low upregulation of *γ*-H2AX in C-cell cultures, which may account for chromosomal instability. In this context, we observed (i) increased G1 cell population in H_2_O_2_-treated C10 cells following Chk1 knockdown and (ii) induction of tetraploidy in C10 cells under acidic conditions, indicating override of the mitotic spindle checkpoint in Chk1-downregulated C10 cells. In addition, 8-OHdG formation was increased in C10 cells with downregulated Chk1 compared to HCEC. Moreover, the 8-OHdG level was even higher in untreated C10 cells than in H_2_O_2_-treated HCEC, suggesting that the base excision repair that was effective in healthy HCEC was defective in C10 cells, which could be a consequence of the low Chk1 levels.

### 4.5. Activated Chk1 Remains Chromatin-Bound following DNA Damage in the Modelled Acute Phase

Shimada et al. and Smits et al. reported that activated Chk1 dissociates from chromatin following DNA damage [20, 22, 23]. Thereby, Chk1 phosphorylation occurs near the sites of DNA damage. In contrast, we observed not only Chk1 dissociation but also association of activated Chk1 in the acute phase. In this context, immobilization of activated Chk1 on chromatin caused defective G2 checkpoint arrest, as the chromatin-bound Chk1 fails to transmit the DNA damage signal to downstream targets [23]. In this context, Scorah et al. identified a PCNA-interacting protein (PIP) box motif in Chk1 that is indispensable for efficient checkpoint responses and release of activated Chk1 from chromatin [42]. It is therefore possible that this motif is not intact in our UC model.

We propose that chromatin association of activated Chk1 further supports the hypothesis that chronic inflammation causes accumulation of DNA damage that is not recognized by the DNA damage response.

### 4.6. Chronic DNA Damage Causes Association of Activated Chk1 on Chromatin, Paralleled by the Accumulation of HATs in the Modelled Quiescent and Active Chronic Phase

We observed chromatin accumulation of Chk1 and activated Chk1 in C10 cells and H_2_O_2_-treated C10 cells compared to H_2_O_2_-treated HCEC accompanied by a switch from cell cycle arrest to increased proliferation, cell cycle arrest override, and apoptosis resistance. This supports the hypothesis that Chk1 chromatin dissociation seems to be essential for proper DNA damage checkpoint activation [23]. Defective Chk1 release from chromatin may enhance the induction of genes that promote cell cycle progression such as c-Myc, c-Jun, c-Fos, *β*-catenin, and TCF4 [5]. In this context, we detected recruitment of the HAT p300/CBP in C10 cells and increased binding of the HATs GCN5 and PCAF and of the oncogenic transcription factors ATF2 and c-Jun, as well of the acetylated histones Ac-H3 and Ac-H3^K9^ in C10 and H_2_O_2_-treated C10 cells compared to HCEC. Nagy and Tora reported on the protumorigenic effects of chromatin-bound GCN5 and PCAF by their functioning as cofactors for proto-oncoproteins, such as c-Myc [43]. We suggest that binding of Chk1 and activated Chk1 on chromatin causes the recruitment of HATs such as GCN5 and PCAF.

Importantly, the chromatin modulation, which was induced following H_2_O_2_ stress in HCEC, is autonomously sustained and enhanced in C10 cells without any exogenous trigger, and this could be the driving force for uncontrolled proliferation. We suggest that the previously found H_2_O_2_-induced ROS generation in C-cell cultures is responsible for this epigenetically regulated signal transduction [5]. Thus, activated Chk1 is not released from chromatin, and H3^T11^ phosphorylation is maintained despite the presence of DNA damage, and this process triggers DNA damage response bypass and features of oncogenic transformation, such as increased proliferation and accumulated DNA damage.

### 4.7. Proposed Model of Dual Chk1 Function in Signal Transduction and Epigenetic Regulation of Transcription (Figure 10)

- On the one hand, Chk1 suppresses JNK activation and signal transduction. Consequently, p21^WAF1^ and *γ*-H2AX levels were reduced, resulting, together with downregulated Chk1, in checkpoint override and, therefore, reduced DNA damage response. On the other hand, enhanced binding of activated Chk1 on chromatin promotes acetylation via HATs and binding of transcription factors, such as c-Jun. Both events resulted in defective DNA damage checkpoint signaling and, thus, in survival and enhanced proliferation of damaged cells.
- In the acute phase, Chk1 and ATF2 dissociate from chromatin following DNA damage, while activated Chk1 accumulates on chromatin and phosphorylates H3 on T11. As a result, GCN5 is recruited and acetylates H3 at K9. Other HATs were recruited, such as PCAF with the consequence of elevated binding of acetylated histones (Ac-H3, Ac-H4) and of c-Jun. We propose that activated Chk1 triggers the recruitment of GCN5 and PCAF to chromatin under acute oxidative stress conditions. Consequently, cells underwent not only cell cycle arrest and apoptosis but also DNA damage response bypass. In quiescent chronic phase, Chk1 and ATF2 again associate with chromatin despite DNA damage, further recruiting p300/CBP and activated Chk1. Thus, the level of GCN5 and Ac-H3^K9^ increased and that of p-H3^T11^ maintained, and further, HATs were recruited (PCAF, ATF2). We suggest that activated Chk1 recruits PCAF to chromatin in quiescent chronic phase. Overall, these chromatin changes result in increased proliferation and undetected DNA damage due to DNA damage response bypass. In the active chronic phase, Chk1 and activated Chk1 levels remained increased on chromatin compared to acute phase. Moreover, we detected increased binding of GCN5, Ac-H3^K9^, Ac-H3, Ac-H4, p300/CBP, c-Jun, and ATF2 compared to quiescent chronic phase. Thereby, activated Chk1 recruits GCN5 to chromatin. As a result, cells underwent reversible cell cycle arrest after which they further proliferate by showing apoptosis resistance due to DNA damage response bypass.

## 5. Conclusions

Chromatin association of activated Chk1 seemed to be linked to defective DNA damage checkpoint signaling in the presence of DNA damage. We propose that subcellular localisation of constitutively activated Chk1 was important for suppression of JNK activation in quiescent chronic phase in vitro and in vivo. Moreover, binding of HATs GCN5, PCAF, and p300/CBP as well as of transcription factors c-Jun and ATF2 promoted features of oncogenic transformation in our experimental model of UC [5]. Overall, on the one hand, chronic H_2_O_2_ exposures caused decreased Chk1 expression and enhanced 8-OHdG formation in C10 cells. This suggests that low Chk1 level led to the bypass of the base excision repair. In addition, override of the G1/S, intra-S, G2/M, and mitotic spindle checkpoints in Chk1-downregulated C10 cells lead to tetraploidy, a sign of chromosomal instability. On the other hand, comet assay analysis of HCEC, C3, and C10 cells gave evidence that DSB repair could be re-activated in C10 cells, presumably by these chromatin changes. Therefore, we propose that Chk1-mediated chromatin modulation induced by DNA damage is responsible for these observations. As Chk1 is bound to chromatin, it fails to transmit the DNA damage signal for proper activation of the G2/M and mitotic spindle checkpoints as well as of the base excision repair. However, DSB repair seemed to be promoted by the chromatin changes. Thus, Chk1 downregulation and activation in quiescent chronic phase of the UC model may promote tumorigenesis, most likely due to impairment of monitoring DNA integrity during replication and mitotic checkpoint failure. We suggest that targeting chromatin-bound activated Chk1, as well as GCN5, PCAF, p300/CBP, c-Jun, and ATF2, might be a novel potential molecular therapy for the prevention of UC-related tumour progression.

## Supplementary Material

Additional file 1. Figure S1. Induction of tetraploidy in checkpoint-deficient C10 cells following acidosis. Cell cycle analysis of HCEC, C5, and C10 cells that were cultured under acidic conditions (10% CO2) with increasing passage.

## Figures and Tables

**Figure 10:**
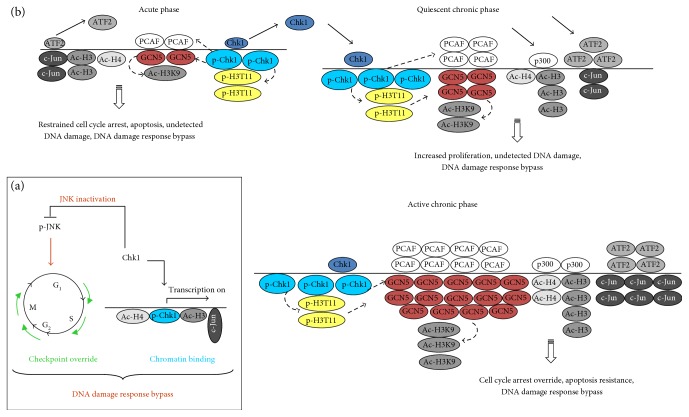
Proposed model for dual function of Chk1 in DNA damage response bypass in experimental UC. (a) On the one hand, Chk1 negatively regulates JNK activation, resulting in checkpoint override and reduced DNA damage response. On the other hand, activated Chk1 remains chromatin-bound and triggers acetylation and binding of transcription factors onto chromatin, leading to induction of proliferative genes and DNA damage response bypass. (b) In the acute phase, Chk1 and ATF2 dissociate from chromatin, while activated Chk1 accumulates on chromatin and phosphorylates H3 at T11. As a result, GCN5 is recruited and acetylates H3 at K9. Moreover, PCAF, Ac-H3, and c-Jun showed elevated chromatin binding. Consequently, cells underwent restrained cell cycle arrest. In the quiescent chronic phase, Chk1 and ATF2 again associate with chromatin despite DNA damage, further recruiting activated Chk1, Ac-H3, GCN5, PCAF, and Ac-H3^K9^, while p300/CBP is firstly recruited to chromatin. These chromatin changes resulted in increased proliferation and undetected DNA damage. In active chronic phase, chromatin binding of Chk1 and activated Chk1 is maintained, while levels of GCN5 Ac-H3^K9^, Ac-H3, Ac-H4, PCAF, p300/CBP, ATF2, and c-Jun are increased. As a result, cells underwent reversible cell cycle arrest and apoptosis resistance. Arrows indicate the appropriate relationship.

## Abbreviations

Ac: Acetylated
ATF2: Activating transcription factor 2
ATR: Ataxia telangiectasia and Rad3 related
AUC: Active ulcerative colitis
CBP: CREB-binding protein
Chk1: Checkpoint kinase 1
CRC: Colorectal cancer
DSB: Double-stranded breaks
H: Histone
H_2_O_2_: Hydrogen peroxide
HAT: Histone acetyltranferase
HCEC: Human colonic epithelial cells
IBD: Inflammatory bowel disease
JNK: c-Jun *N*-terminal kinase
PCAF: P300/CBP-associated factor
QUC: Quiescent ulcerative colitis
SSB: Single-stranded breaks
UC: Ulcerative colitis
UC-CRC: Ulcerative colitis-related colorectal cancer.
