# Supplementary figures and images for: Chk1 Promotes DNA Damage Response Bypass following Oxidative Stress in a Model of Hydrogen Peroxide-Associated Ulcerative Colitis through JNK Inactivation and Chromatin Binding
Source: Oxid Med Cell Longev. 2017 Jun 7;2017:9303158. doi: 10.1155/2017/9303158 (PMC5478872; doi:10.1155/2017/9303158)

## Slide 1
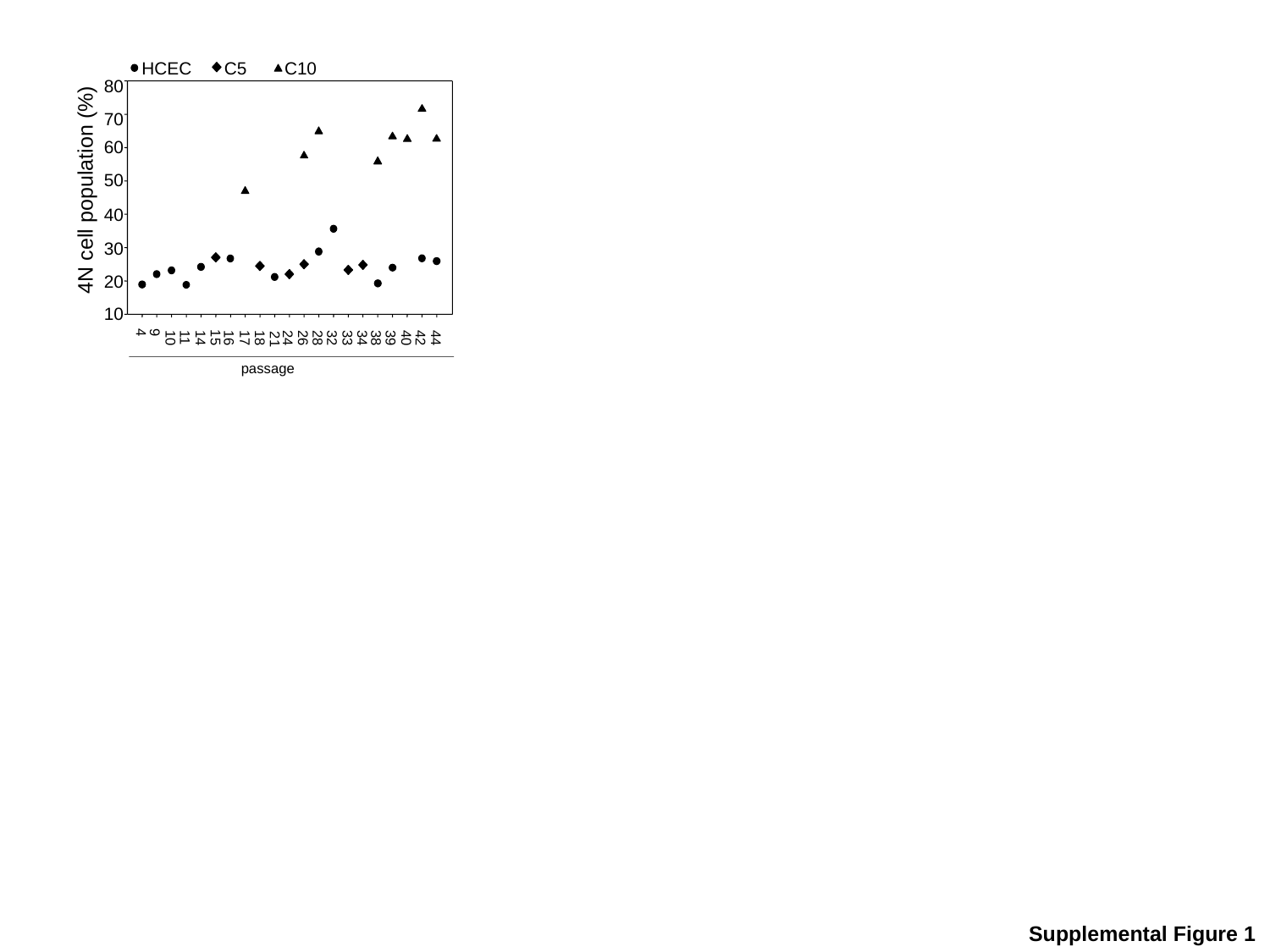

C5
C10
HCEC
80
70
60
50
4N cell population (%)
40
30
20
10
9
4
15
10
14
17
18
24
26
28
33
38
39
40
44
11
34
42
16
32
21
passage
Supplemental Figure 1

Supplement: Supplementary file 1 — Additional file 1. Figure S1. Induction of tetraploidy in checkpoint-deficient C10 cells following acidosis. Cell cycle analysis of HCEC, C5, and C10 cells that were cultured under acidic conditions (10% CO2) with increasing passage. [file 9303158.f1.pptx]
